# Massage manipulation and progression of osteosarcoma, does it really correlate: a combination of prospective and retrospective cohort study

**DOI:** 10.1038/s41598-023-45808-7

**Published:** 2023-10-29

**Authors:** I Wayan Arya Mahendra Karda, Wan Faisham Wan Ismail, Achmad Fauzi Kamal

**Affiliations:** 1 Department of Orthopaedics and Traumatology, Cipto Mangunkusumo Central General Hospital, Faculty of Medicine Universitas Indonesia, Pangeran Diponegoro Street Number 71, Central Jakarta, Jakarta, 10430 Indonesia; 2 Department of Orthopaedics, Universiti Sains Malaysia, Kubang Kerian, Kelantan Malaysia

**Keywords:** Health care, Oncology

## Abstract

In Indonesia, the challenge of osteosarcoma progression is further worsened by patients' dependence on traditional massage therapy, low socio-economy, and educational status. This study aims to analyze the differences in the characteristics, laboratory findings, surgery techniques, degree of histopathological necrosis, and metastasis between osteosarcoma patients with and without prior massage manipulation therapy. This research is an analytical observational study with a prospective and retrospective cohort design. Patients were treated and followed for one year to evaluate the occurrence of metastasis. Prospective data was collected through interviews, and secondary data was collected from the patient's medical record. Of 84 subjects analyzed, 69% had a history of massage. There was an increase in LDH and ALP in patients with massage manipulation (*p* = 0.026). The median time to metastasis from baseline in the massage group (4 months) was statistically significant compared to the non-manipulation group (12 months) (*p* < 0.0001). This research found that massage therapy significantly increases LDH and ALP levels, making amputations more likely to be performed and a higher risk of metastasis that lowered the survival rate. The onset of metastasis was three times faster in patients with prior massage therapy. Therefore, we strongly recommend against massage manipulation therapy in osteosarcoma patients.

## Introduction

Osteosarcoma is a malignant bone tumor originating from primitive mesenchymal cells that produce bone and can differentiate into osteoblasts, chondroblasts, and fibroblasts^1^. It is the most common primary malignant bone tumor in children and young adults, with a bimodal age distribution in the second and seventh to eighth decade of life^2–4^. A study by Kamal et al.^5^, found 219 cases of osteosarcoma at the National Central General Hospital Jakarta, Indonesia, within a period of 13 years (1995–2007) with a prevalence of 16.8 cases annually. This figure increased to 373 patients or about 19 per year for 20 years (1995–2014)^6^.

Treatment for osteosarcoma is generally a combination of chemotherapy and surgery^7^. Until 30 years ago, the five-year survival rate for osteosarcoma was less than 20 percent. With the advent of chemotherapy and the development of surgical techniques, the long-term survival rate of osteosarcoma patients has increased to 60–70%^3,7^. Since the introduction of chemotherapy, limb salvage surgery can be performed in 85–97% of cases^8^. Several factors have been associated with osteosarcoma's poor prognosis, including increased alkaline phosphatase level serum, large tumor size, low tumor necrosis ratio, and metastasis^7,9^.

In the early stage, the non-specific nature of the pain causes many patients to seek alternative treatment before the diagnosis is confirmed, namely massage therapy^7,8^. In many Asian countries, massage therapy is famous for various health problems^3^. Based on the 2018 Indonesian Research Data Registry, around 31% of the Indonesian population used traditional health services, including massage for fractures, reflexology, acupuncture, chiropractic, and so on^8^.

Previous studies have stated that massage manipulations performed on tumor areas are thought to form micrometastasis due to hypervascularization, which results in poor prognosis^3,4,7^. Local spread of tumors due to massage therapy makes limb salvage surgery difficult and is associated with a poor survival rate^3^. To date, not many studies have been conducted to determine the effect of massage therapy on osteosarcoma patients^4,7^. Some of the existing studies include in vivo studies and a combination of retrospective and prospective cohorts. In Indonesia, the challenge of osteosarcoma progression is amplified by patients' dependence on traditional therapy and low socioeconomic levels. Most osteosarcoma patients come at the more advanced stages^8,10^. This study aims to evaluate the effect of massage manipulation therapy and osteosarcoma progressivity.

## Methods

This study was an observational analytic study with a prospective and retrospective cohort design. This study was conducted at a tertiary National Central General Hospital in Jakarta, Indonesia, from January 2021 to October 2022. Patients diagnosed with osteosarcoma between 2017 and 2022 were included. The patient must be: (1) an osteosarcoma patient diagnosed based on clinical-pathological conference recommendations; (2) had received neoadjuvant chemotherapy; (3) with complete clinical, laboratory, and histopathological data; and (4) had undergone surgical treatment at our institution. We excluded patients with axial or low-grade osteosarcoma, patients’ with incomplete data, and patients’ that were treated in another hospital.

There were two types of data in this study namely prospective and retrospective data based on the time of diagnosis. Retrospective secondary data was collected from the medical records in patients’ before the enrollment of the study (between January 2017 and January 2021). Prospective data collection was conducted by interview, physical, laboratory and radiological examination of the research subjects in patients after the study started (after January 2021). Total sampling method was used in this prospective data collection that met the inclusion criteria. The patients’ were diagnosed and treated accordingly by our senior Orthopaedic oncology specialist (AFK). Those patients’ were then evaluated regularly for metastasis until 1-year follow-up. Variables such as age, gender, symptoms duration, tumor size, tumor location, laboratory results (lactate dehydrogenase and alkaline phosphatase level), types of surgery, degree of histopathological necrosis, metastasis at initial diagnosis, metastasis following treatment, and metastases rate were recorded after a written informed consent.

History of massage manipulation therapy was recorded retrospectively from medical records and prospectively by interviewing the research subjects during the first polyclinic visit. Massage manipulation in this study was defined as systematic mechanical compression, of the tumor area ≥ 1 time before coming to a health facility, performed by traditional healers with certain intensity, direction, rhythm using/without assistive devices. The patients who were being massaged outside the tumor area were considered not being manipulated before initial diagnosis. The patients were also grouped according to the frequency as having < 3 or ≥ 3 times of massage manipulation before the initial diagnosis.

All patients were evaluated during the initial diagnosis for pulmonary metastasis by plain film and thorax computed tomography (CT scan). Evaluations were then performed at one year or early (if there were signs of pulmonary metastasis) after definitive surgery to determine the response of treatment.

All patients must received neo adjuvant chemotherapy according to our instititional protocol that consisted of Cisplatin 60 mg/m^2^, Ifosfamide 3000 mg/m^2^ per day, Uromitexan 3000 mg/m^2^ per day and Adriamycin 25 mg/m^2^ per day. The protocol was given in one to three cycles for 6 to 8 weeks according to the patients’ condition. After completion of neo adjuvant chemotherapy, the patient’s underwent limb salvage surgery or amputation accordingly. Patients underwent adjuvant chemotherapy 2 to 4 weeks after local control of the tumor for 6 weeks. The protocol for adjuvant chemotherapy included Cisplatin 60 mg/m^2^, Ifosfamide 3000 mg/m^2^ per day, and Adriamycin 25 mg/m^2^ per day.

The response to neo adjuvant chemotherapy was evaluated according to Huvos scale in which grades I to IV denoted 0% to 50%, 51% to 90%, 91% to 99% and 100% necrosis, respectively. Grades III and IV were defined as a good reponder while grades I and II were considered as poor responder^7^.

Statistical analysis using SPSS was done to determine the association between massage therapy and clinical variables, laboratory, and metastasis. Categorical data were analyzed using the Chi-square test or Fisher exact test and numerical data using the Independent T-test or Mann Whitney. The research is conducted in accordance with the Declaration of Helsinki with the assurance of the subject’s health and rights.

### Consent to participate

Informed consent was obtained from all individual participants included in the study.

### Ethics Approval

This research has received ethical approval from The Ethics Committee of the Faculty of Medicine, Universitas Indonesia–Cipto Mangunkusumo Hospital. (Protocol number 21-02-0171). This research was registered in Clinical Trial Registry with identifying number of research registry 9601.

## Results

There were 84 subjects met the inclusion and exclusion criteria. Table 1 presents the subjects' demographics in both study groups. There was no statistical difference in subject characteristics (age, gender, symptom duration, tumor sizes, tumor location, and type of surgery) between groups. Laboratory results tend to increase in those who received massage therapy compared to those who did not (RR 3.057, 95% CI 1.105–6.853, *p* = 0.026). All patients had adequate surgical margin resection intra-operatively (clear margin with distance of ≥ 2 mm).

**Table 1:** Subject characteristics and osteosarcoma progressivity variable.

Characteristics	Massage therapy	Without massage	RR (95% CI)	*P* value
Age (years)^a^	15.5 (5–47)	16 (5–65)		0.641*
Gender^b^				
Male	35 (60.3%)	12 (46.2%)	1.775 (0.69–4.52)	0.226†
Female	23 (39.7%)	14 (53.8%)		
Symptoms duration (months)^a^	5 (1–24)	4.5 (1–18)		0.682*
Tumor sizes (cm)^a^	3.18 (0.16–16.24)	1.91 (0.64–29.30)		0.423*
Tumor locations^b^				
Near the knee joint	45 (77.6%)	21 (80.8%)	1.182	0.742†
Far from the knee joint	13 (22.4%)	5 (19.2%)	(0.472–2.614)	
Laboratory results^b^				
Increased	45 (77.6%)	15 (57.7%)	3.057	**0.026**†
Not increased	13 (22.4%)	11 (42.3%)	(1.105–6.853)	
Types of surgery^b^				
Amputation	31(53.4%)	9 (34.6%)	1.461	0.110†
LSS	27 (46.6%)	17 (65.4%)	(0.177–1.203)	

Significant values are in bold.

^a^Median (Min–Max).

^b^Frequencies (%).

*Comparison were made using Mann–Whitney test.

†Comparison were made using Fisher exact test.

Amputation is the type of surgery primarily performed on subjects with massage therapy (53.4%). Limb salvage surgery was performed in 65.4% of subjects who did not receive massage therapy. Table 2 presents osteosarcoma progression between massage and non-massaged groups. Subjects with massage therapy had a lower degree of histopathological necrosis or poor responders of 93.1%, higher than the group of subjects who did not receive massage therapy, which was 76.9%. 55.2% of subjects did not have metastases at the time of initial diagnosis in the massage therapy group.

**Table 2:** Osteosarcoma progressivity variable in between study groups.

Characteristics	Massage therapy	Without massage	RR (95% CI)	*P* value
Degree of necrosis^b^				
Poor Responder	54 (93.1%)	20 (76.9%)	0.247	0.063†
Good Responder	4 (6.9%)	6 (23.1%)	(0.063–0.967)	
Lung metastasis at initial diagnosis^b^				
Metastasis	26 (44.8%)	8 (30.8%)	1.758	0.225†
Without metastasis	32 (55.2%)	18 (69.2%)	(0.686–4.873)	
Lung metastasis following treatment^b^				
Metastasis	45 (77.6%)	18 (69.2%)	1.538	0.414†
Without metastasis	13 (22.4%)	8 (30.8%)	(0.546–4.338)	
Time from initial diagnosis until metastases (months)^a^	2 (0–22)	12 (0–32)		< 0.0001*

Significant values are in bold.

^a^Median (Min–Max).

^b^Frequencies (%).

*Comparison were made using Mann–Whitney test.

†Comparison were made using Fisher exact test.

On the other hand, 69.2% of subjects had no metastases at initial diagnosis in subjects who did not receive massage therapy. Pulmonary metastasis following treatment occurred in subjects with massage therapy in 45 patients (77.6%), compared with 18 patients (69.2%) in subjects who did not receive massage therapy. The degree of necrosis (*p* = 0.063), lung metastasis at initial diagnosis (*p* = 0.225), and lung metastasis following treatment (*p* = 0.414) were not correlated with massage therapy. However, there is a significant correlation between the rate of metastasis and massage therapy (*p* =  < 0.0001). The median time to metastases from initial diagnosis in the massage therapy group was 4 (2—11) months, whereas in the non-massage group, received massage therapy was 12 (8–32) months.

In this study, massage manipulation had no statistical effect on the degree of histopathological necrosis using the Fischer test (*p* = 0.063). However, massage manipulation had a risk for poor responders because the relative risk range indicated that massage manipulation was a causative factor (RR = 0.247; 95% CI = 0.063–0.967).

The frequency or number of massages was divided among all the patients who underwent massage therapy. Eighteen patients underwent massage more than equal to 3 times and 26 patients who underwent massage less than 3 times. The characteristics and variables of osteosarcoma progression in massage therapy subjects and those who did not receive massage therapy are shown in Table 3.

**Table 3:** Frequency of massage manipulation on laboratory results and metastasis.

Massage frequency	Increased lab results^b^	Lab results not increased^b^	RR (95% CI)	*P*	Rate of metastasis from initial diagnosis in months^a^	*P*
< 3 times	17 (56.7%)	9 (64.3%)	0.726 (1.196–2.693)	0.632†	7 (0–22)	0.287*
≥ 3 times	13 (43.3%)	5 (35.7%)			6 (0–12)	

^a^Median (Min–Max).

^b^Frequencies (%).

*Comparison were made using Mann–Whitney test.

†Comparison were made using Fisher exact test.

## Discussion

In this study, 45 subjects from 58 patients with a history of massage therapy had significantly increased laboratory results (75.0%). Lactate dehydrogenase (LDH) plays a role in the interconversion of lactate and pyruvate, providing NAD + for the continuation of glycolysis in active muscle^6,11^. The enzyme alkaline phosphatase (ALP) is widely distributed in the liver, bones, kidneys, gastrointestinal tract, and placenta and is excreted from the body via the liver and biliary system^12^. An increase in LDH serum level is usually accompanied by increased of ALP and associated with poorer prognosis for osteosarcoma^13^. In this study, after analyzing the effect of massage therapy on increasing laboratory results, further analysis was carried out to determine whether the frequency of massage affected the results. However, the result was considered not statistically significant. Not many studies have analyzed the relationship between massage therapy and an increase in laboratory results. The results of this study are in accordance with study by Wu et al. which stated that massage therapy increased LDH and ALP. Other study by Fu et al., also found that an increase in LDH serum was associated with a decrease in event-free survival (EFS) and recommended that LDH serum be used as a prognostic biomarker for osteosarcoma patients. Moreover, in the study by Bacci, the increase in LDH serum was significantly associated with relapse, rate of recurrence, and significantly poorer overall survival^14^.

Amputation was the type of surgery primarily performed on subjects with massage therapy (53.4%). Limb salvage surgery was performed in 65.4% of subjects who did not receive massage therapy. However, there was no statistically significant result between massage manipulation and types of surgery. This result is supported by study from Wu et al.^7^ which showed local recurrences were more common in patients receiving manipulation therapy. Before the introduction of neo adjuvant chemotherapy, amputation and disarticulation were the most common treatments in osteosarcoma patients with a five-year survival rate of only 20 percent^15^. A meta-analysis study by Papakonstantinou et al.^16^ in 2884 osteosarcoma patients, found that the five-year survival rate of patients undergoing LSS is higher than amputation.

This study found that patients with massage manipulation had the most significant proportion of pulmonary metastases after treatment, as many as 45 patients (71.4% of the total patients with massage manipulation). Despite the large proportion of patients with massage manipulation in osteosarcoma patients with pulmonary metastases, these results did not show a significant relationship between massage manipulation and lung metastases after treatment.

From this study, the median time to metastasize since initial diagnosis in the massage-manipulation group was 4 months, while in the non massage-manipulated group, it was 12 months. When the Mann–Whitney test was performed, there was a significant difference in the rate of metastasis between the two groups, wherein for patients with osteosarcoma, massage manipulation increased the rate of metastasis compared to those who did not receive massage manipulation (*p* < 0.0001). These results were then analyzed to determine the relationship between massage frequency in the massage manipulation group and the rate of metastasis. It was found that there was no significant difference in the rate of metastasis of the two massage frequency groups < 3 times compared to ≥ 3 times (*p* = 0.287). Based on research by Wu et al., it was found that massage manipulation increased the incidence of metastases compared to those who did not receive massage manipulation (*p* < 0.001). However, unlike this study, there was no significant difference in the rate of metastasis in the two groups (*p* = 0.119)^7^.

In this study, massage manipulation increased the rate of metastasis compared to those who did not receive massage manipulation (*p* < 0.0001). Massage manipulation is thought to increase the risk of early metastasis in osteosarcoma patients. The presence of metastases in osteosarcoma patients affects survival. Five-year survival of patients with metastases is 63% compared to 71% of patients without metastases^17,18^. The degree of histopathological necrosis of osteosarcoma in this study was divided into two groups: poor responders (Huvos I-II) and good responders (Huvos III-IV). This study shows that massage manipulation has no statistical effect on the degree of histopathological necrosis. This result is in line with a study by Wu et al.^7^ which stated that massage manipulation did not significantly affect the degree of histopathological necrosis.

From this study, most patients (88.1%) had a poor response to chemotherapy (poor responders). These results are similar to other studies by Prabowo et al. and Chui et al. which stated that most high-grade osteosarcoma patients had a poor response to chemotherapy (76.6 and 60%)^19,20^.

Conventional osteosarcoma with chondroblastic, osteoblastic, fibroblastic, telangiectatic and small-cell subtypes tend to metastasize and are known as high-grade osteosarcoma. High-grade surface osteosarcoma and secondary osteosarcoma are also high-grade tumors that tend to metastasize mainly to the lungs^20^. Histological type is known to influence patient outcomes. Compared to other histological types, osteosarcoma with fibroblastic differentiation has been associated with good prognosis. Worse outcomes were found in chondroblastic type according to existing studies. Telangiectasis osteosarcoma is known to have good sensitivity to chemotherapy due to the high cell cycle and increased tumor vascularity^19,21^.

In this study, the samples used were patients with high-grade osteosarcoma and excluded patients with low-grade tumors and osteosarcoma located in the axial bone. This is done to reduce potential confounding factors caused by different histopathological types of osteosarcoma.

In this study, the results obtained for poor responders were higher than for good responders. These results are pretty different compared to the study by Bacci et al. which stated that an excellent response to chemotherapy was obtained at 50.1–55.6%. There were differences in patient characteristics and the use of methotrexate in the study. Differences in chemotherapy regimens are thought to be one of the factors influencing differences in histopathological responses after chemotherapy^20^. Several randomized controlled trials state high-dose methotrexate as the essence of neoadjuvant chemotherapy^22^.

Several mechanism have been proposed to explain how massage therapy induce metastasis. First, mechanical force in message therapy is thought to induce lymphatic spreading. Diaz et al.^23^ have reported mechanical transport of epithelial cell to axillary lymph node due to cell lesions from mechanical force prior to surgery. Second, increased MMP expression due to mechanical force. Several studies have shown that MMP is an extracellular matrix factor that have a role in tumor cell metastasis^24,25^. High expression of MMP have been reported to have an association with poor prognosis and metastasis risk^26–29^. In a study by Wang et al.^4^, elevated serum level of MMP3 and MMP9 was found in message therapy group. Message therapy was also shown to improve circulation^30^, promote and stimulate neovessel formation^31–33^, and increase the expression of human KDR detected in xenograft injected tumors which increased the risk of metastasis due to increased blood flow in tumors^4^.

This study has several strengths and limitations. This study has successfully controlled confounding variables such as age, gender, symptom duration, tumor sizes, tumor location, and type of surgery which was reported to have a high prognostic value in osteosarcoma patients^34–37^. The limitation of this study is the possibility of recall bias of the massage manipulation frequency, especially in 12 subjects who did not recall the massage manipulation frequency. Furthermore, we did not analyze the subgroup of the massage manipulation, including types, techniques, mechanical force, or the quality of the massage. Long-term follow-up after 1 year was not conducted in this study while several studies have reported the survival rate of high grade osteosarcoma to be 57%–67.7%^38–40^. Thus, we cannot observe the progressivity and prognosis of osteosarcoma.

## Conclusion

There were no significant differences in the characteristics, surgery techniques, and histopathological necrosis degree in osteosarcoma patients who received and did not receive prior manipulation therapy. There were significant differences in laboratory findings between the two groups. Although there was a higher proportion of metastases in the manipulation group, there was no significant difference in terms of metastases at initial diagnosis and post-treatment, but the time to metastases was significantly faster in patients with prior manipulation therapy.

## Data Availability

All the data are available from the corresponding author and can be accessed via corresponding email after clearly stating the intention and permission to conduct research that requires our data.
